# Co-modulation of finely tuned high-gamma band activity across hemispheres in Parkinson’s disease

**DOI:** 10.1016/j.clinph.2013.10.001

**Published:** 2014-04

**Authors:** Hayriye Cagnan, Andrea A. Kuhn, Peter Brown

**Affiliations:** a Department of Clinical Neurology, University of Oxford, John Radcliffe Hospital, West Wing Level 6, OX3 9DU Oxford, UK; b Department of Neurology, Campus Virchow Klinikum, Charité-University Medicine Berlin, 13353 Berlin, Germany

**Keywords:** Parkinson’s disease, Deep brain stimulation, Gamma frequency band, Co-modulation

## Abstract

**Objective:**

Dopaminergic treatment of patients with Parkinson’s disease can be associated with a finely tuned high-gamma (FTG) band oscillation between 60 and 90 Hz in the basal ganglia (BG) nuclei. This activity, together with suppression of excessive neural synchrony in the beta frequency band (15–30 Hz), is hypothesized to contribute to the pro-kinetic effects of dopaminergic medication. Beta activity, which high-gamma replaces, has been shown to be bilaterally coherent, but whether sub-cortical FTG activity is in some way linked across hemispheres remains unknown.

**Methods:**

We analyse bilateral local field potential activity recorded from the subthalamic nuclei of 10 patients on dopaminergic medication while at rest, following surgery for deep brain stimulation.

**Results:**

In three of these patients FTG activities were coherent across the hemispheres. Short duration (⩽18 s) bilateral amplitude and frequency co-modulation were recorded in all subjects, although the two phenomena were independent in time and independent of similar episodes in other frequency bands.

**Conclusions:**

FTG co-modulation can be independent of movement and of co-modulation episodes at other frequencies.

**Significance:**

Relatively independent organisation of processing through amplitude and frequency modulation within different frequency bands potentially affords opportunities for functional segregation and multiplexing of processing streams within the BG.

## Highlights

• Episodes of amplitude and frequency co-modulation occur in the high-gamma frequency band between hemispheres in patients with Parkinson’s disease.
• Co-modulation is spontaneous and can be independent of voluntary movement.
• Co-modulation occurs without phase delay between the hemispheres and may reflect shifts in arousal.

## 1 Introduction

Use of functional neurosurgery for treatment of movement disorders such as Parkinson’s disease (PD) has made exploration and characterisation of basal ganglia (BG) pathophysiology possible (Hammond et al., 2007). Single unit neuronal and local field potential (LFP) activities can be captured from electrodes implanted in BG surgical targets like the subthalamic nucleus (STN) and globus pallidus. Such recordings have been invaluable in the characterisation of human BG activity during different medication and behavioural states (Jenkinson and Brown, 2011).

Loss of dopamine in the substantia nigra pars compacta of patients with PD is associated with excessive synchronization of neural activity in the beta frequency band (15–30 Hz). This excessive activity is coherent ipsilaterally across the BG and between the BG and ipsilateral motor cortex (Brown et al., 2001, Marsden et al., 2001). Additionally, it is coherent between the two STNs, when patients with PD are withdrawn from their dopaminergic medication and are recorded at rest (de Solages et al., 2010).

**Fig. 1:**
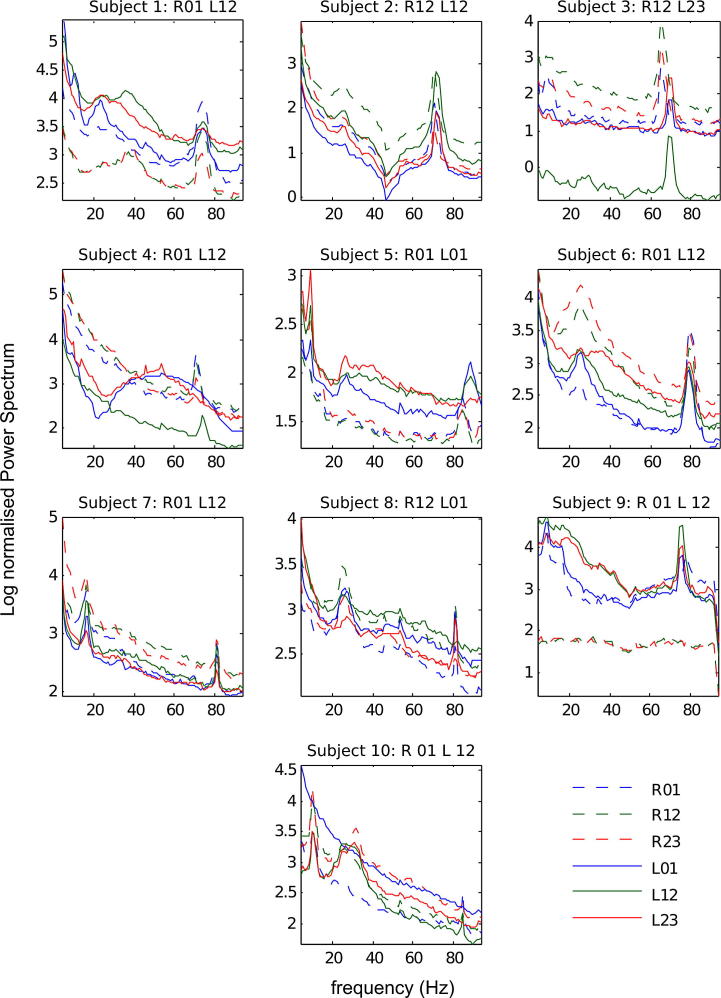
Log normalized LFP power spectral density for all subjects from the three DBS contact pairs 0–1, 1–2, and 2–3, left and right STN. Contact pair analysed for each subject is indicated in the title.

When PD patients are administered dopaminergic medication, excessive beta synchrony is reduced and synchronization of neural activity at higher frequencies is observed, for example as finely tuned peaks between 60 and 90 Hz in the high-gamma frequency band (FTG) (Fig. 1) (Brown, 2003, Brown et al., 2001). It has been suggested that this high-gamma activity is pro-kinetic, particularly as it increases contralateral to the side of voluntary movement (Androulidakis et al., 2007a, Cassidy et al., 2002, Kühn et al., 2006a). However, it remains unknown whether bilateral coherence observed in the beta frequency band in the STN extends to other frequency bands such as the high-gamma frequency band after dopaminergic treatment, and, if it does, what its function might be. Here, we address this question through the analysis of LFP activity recorded bilaterally from the subthalamic nuclei of patients with PD undergoing surgery for deep brain stimulation (DBS).

## 2 Methods

### 2.1 Patients and LFP recordings

All patients gave their informed consent to take part in the study, which was approved by the local ethics committees at our recording sites in London, Rome and Berlin. Our aim was to test the independence or otherwise of discrete high-gamma band peaks in the LFPs, recorded at rest bilaterally from the two STNs. We thus screened archival data from 25 PD patients who had undergone bilateral implantation of DBS electrodes into the STN, as a prelude to therapeutic high frequency stimulation for advanced idiopathic PD with motor fluctuations and/or dyskinesias. Only data obtained while patients were on levodopa (L-DOPA) was considered so as to maximise the incidence and amplitude of the high-gamma band activity (Alegre et al., 2005, Alonso-Frech et al., 2006, Androulidakis et al., 2007a, Brown et al., 2001, Cassidy et al., 2002, Devos et al., 2006, Fogelson et al., 2005). We identified recordings from 10 patients with bilateral discrete high-gamma band peaks for further analysis (Fig. 1 and Table 1). Techniques to target and implant electrodes in the STN have previously been described Foltynie and Hariz (2010). Microelectrode recordings were made during surgery in Rome and Berlin. The permanent quadripolar macroelectrode used was model 3389 (Medtronic Neurologic Division, Minneapolis, MN, USA) featuring four platinum-iridium cylindrical surfaces. Its contacts are numbered 0, 1, 2, and 3, with 0 being the most caudal and contact 3 being the most cranial. Localisation was supported intra-operatively by microelectrode recordings in two centres, the effects of direct stimulation and by immediate post-operative stereotactic imaging. DBS electrode extension cables were externalized through the scalp to enable recordings prior to connection to a subcutaneous DBS pacemaker, implanted in a second operative procedure up to seven days later. Mean percentage improvement in the motor section of the Unified Parkinson’s Disease Rating Scale (UPDRS; (Fahn and Elton,1987)) on treatment with L-DOPA was 61 ± (SD) 15% (*p* < 0.001, paired *t*-test between ON and OFF L-DOPA scores), indicating very good responsiveness to L-DOPA by our study participants, while improvement in the motor section of the UPDRS off L-DOPA, on DBS was 50 ± (SD) 11% with respect to off L-DOPA, off DBS (*p* < 0.0025, paired *t*-test between ON and OFF DBS scores), supporting satisfactory DBS electrode placement.

**Table 1:** Patient information on age, gender, disease duration, symptoms, and UPDRS scores. Total L-DOPA equivalent dose was estimated according to (Tomlinson et al., 2010). Case 1 was operated at the National Hospital for Neurology and Neurosurgery in London, Cases 2 and 3 were operated at the Centro Traumatologico Ortopedico, A. Alesini Hospital, in Rome, and cases 4–10 were operated on at the Charité, Berlin. Data obtained from subject 1 has been previously published in (Fogelson et al., 2005), subject 4 in (Kühn et al., 2006b), subject 6 in (Doyle et al., 2005, Kühn et al., 2006a, Kühn et al., 2006b), subject 7 in (Kühn et al., 2009), and subject 10 in (Kühn et al., 2006a, Kühn et al., 2006b, Kühn et al., 2009, Trottenberg et al., 2006).

	Age/gender	Disease duration (years)	More affected side	Predominant symptoms	Pre-op UPDRS OFF/ON	Post-op UPDRS – DBS OFF/ON (while OFF med)	Total l-DOPA equivalent dose/day (mg)
1	46/m	13	Right	Dyskinesias on	63/08	61/21	1990
2	54/f	6	Right	Dyskinesias on	48/29	46/31	1320
3	59/f	13	Left	Dyskinesias on	27/08	26/13	300
4	51/m	6	Left	On–off fluctuations, Dyskinesia on	40/15	35/22	1320
5	67/f	12	Right	Bradykinesia off, Dyskinesias on, Freezing	57/29	N/A	1370
6	62/f	12	Right	Bradykinesia off, Dyskinesias on	51/16	54/25	1080
7	79/m	7	Right	Bradykinesia off, Tremor off	30/07	30/14	540
8	57/f	7	Right	Wearing off	33/13	35/19	510
9	69/m	16	Left	Tremor	47/26	30/14	1000
10	57/m	17	Left	Tremor	39/18	44/8	600

LFP recordings were made 3–6 days after surgery. Recordings were made on average 60 min following L-DOPA administration, after overnight withdrawal of anti-parkinsonian medication. L-DOPA dosage was the same as the patients’ usual first morning dose (median dose 200 mg, range 100–300 mg). Improvement with medication was confirmed through assessment of finger tapping, wrist rigidity and tremor (using the corresponding items of the motor UPDRS). Subjects rested in a chair with their eyes open. They were asked to remain quiet and still, and rest was confirmed by visual inspection. Periods of dyskinesia, tremor or voluntary movement detected by the examiner on visual inspection were noted and excluded from further analysis. LFPs were recorded bipolarly from each DBS electrode as contact pairs 0–1, 1–2, and 2–3. Signals were band-pass filtered between 1 and 250 Hz. Signals were amplified (×50,000) and digitised in two ways: (1) In nine cases (Rome and Berlin) we used a custom-made, 9 V battery-operated high-impedance amplifier (INA128 instrumentation amplifier, Texas Instruments, Inc., Dallas, TX, USA). These signals were sampled at 1 kHz (with two exceptions which were at 526 and 625 Hz). They were either digitised through a 1401 A/D converter (Cambridge Electronic Design, Cambridge, UK) onto a computer using Spike2 software (Cambridge Electronic Design; 8 cases in Berlin) or through an A-D card (PCM-DAS16S; Computer Boards, Massachusetts, USA; 2 cases in Rome) onto a portable computer using custom written software. (2) One subject (London; case 1) was recorded using a D360 amplifier (Digitimer Ltd, Welwyn Garden City, Hertfordshire, UK), sampled at 2 kHz and recorded through a 1401 A/D converter onto a computer using Spike2 software. Given the differences in recording techniques we only contrasted normalised measures (coherence and correlation) and not absolute power levels in later analysis. 238 ± [SEM] 50 s of data were available for analysis in each patient.

### 2.2 Data analysis and statistics

LFP recordings were analysed offline using MATLAB. All recordings were down-sampled to 512 Hz and filtered using a sixth order notch filter with a pass-band ripple of 1 dB in order to suppress 50 Hz power line artifact.

#### 2.2.1 Power spectral analysis

Power spectral density was calculated using short-time Fourier transform with a Hamming window of 1 s and 75% overlap.

#### 2.2.2 Contact pair selection

An expert neurosurgeon or neurologist specialising in deep brain stimulation who was blinded to the electrophysiological results determined which electrode contacts were in the STN from post-operative MRI. This afforded up to three bipolar contacts that had at least one contact within the STN per electrode (13 STN with two bipolar pairs and 5 STN with three bipolar pairs). From these contact pairs we then selected the one exhibiting the highest gamma (60–90 Hz) power for further analysis, as this provides further evidence that at least one of the contacts lies in STN (Fogelson et al., 2005, Trottenberg et al., 2006). The post-operative MRI was unavailable in case 9, so we took the contact pair with the highest gamma power, as before. Only one channel per electrode was subsequently analysed in terms of cross-hemisphere co-modulation, regardless of the frequency band of interest (Fig. 1).

#### 2.2.3 Coherence

Coherence between bilateral LFP recordings was estimated using Welch’s averaged modified periodogram method with non-overlapping Hamming windows (Rabiner and Gold, 1975, Welch, 1967). Window length was set to 1 s giving rise to a frequency resolution of 1 Hz. The threshold for significant coherence (*p* = 0.05) was determined using a method based on surrogate time series. For each side, 1000 surrogate time series were generated by computing the Fourier Transform of the original time series, resampling phase without replacement while keeping the modulus of the time series unchanged and applying the inverse Fourier transform in order to return to the time domain. Coherence was then estimated between the bilateral surrogate time series and the threshold for significant coherence was set at 95th percentile of the coherence values obtained from the surrogate pairs.

#### 2.2.4 Amplitude envelope and instantaneous frequency

The LFP was pass-band filtered in both the forward and reverse directions, to achieve zero phase distortion, using a tenth order Butterworth filter with a pass band of ±5 Hz around the average FTG peak frequency. If the FTG peak frequencies were more than 1 Hz apart on the two sides the filter’s pass-band window was chosen so that both peaks were included in a 10 Hz wide pass-band.

The amplitude-envelope and instantaneous frequency of the filtered time series were estimated using the Hilbert Transform of the signal. The amplitude envelope of the FTG oscillation was computed using:

$$A(x)=\sqrt{x{\left(t\right)}^{2}+H{\left(x(t)\right)}^{2}}$$

where *x*(*t*) is the pass-band filtered time series and *H*(*x*(*t*)) is the Hilbert Transform of the pass-band filtered time series. The instantaneous frequency of the FTG oscillation was computed by differentiating the unwrapped phase of the analytic signal constructed from the Hilbert Transform of the pass-band filtered time series (real part of the analytic signal is equivalent to *x*(*t*) and the imaginary part of the analytic signal is equivalent to the Hilbert Transform of *x*(*t*)). Instantaneous phase of the analytic signal was obtained using:

$$\phi \left(t\right)=\text{arctan}\left(H(x(t))\text{,}x(t)\right)$$

Bilateral co-modulation of the amplitude-envelopes and instantaneous frequencies in the high-gamma band was estimated by computing the maximum cross-correlation level between bilateral amplitude-envelopes and bilateral instantaneous frequencies over 1 s long 87.5% overlapping windows. If the maximum cross correlation occurred at a lag greater than 30 ms or less than −30 ms, the cross-correlation level was set to zero, so as to focus on relatively direct interactions between the two STNs and the effects of bilaterally synchronous inputs. Relaxing the limit on lags to ±150 ms made less than 3% difference in amplitude-envelope and instantaneous frequency co-modulation (results not shown). Threshold for significant cross-correlation was calculated utilizing a method based on surrogate time series. For each side, 1000 surrogate time series were generated by computing the Fourier Transform of the original time series, resampling phase without replacement while keeping the modulus of the time series unchanged and applying the inverse Fourier transform to return to the time domain. The amplitude-envelope and instantaneous frequency of the shuffled time series were calculated as before. Threshold for significant correlation was set at the 95th percentile of the cross-correlation values obtained from bilateral amplitude-envelopes or instantaneous frequencies of the surrogate pairs.

#### 2.2.5 FTG co-modulation dependence on other frequency bands

In order to test whether co-modulation of amplitude-envelopes and instantaneous frequency in the high-gamma band was dependent on bilateral changes in other frequency bands, we calculated the number of time segments with amplitude or frequency co-modulation in the high-gamma band, which also contained co-modulation of amplitude or frequency in the theta/alpha band (4–14 Hz), the beta band (15–30 Hz) or the low-gamma band (31–48 Hz) (Fig. 1). Significance of these coincidence rates was tested by shuffling the 1 s long time segments 1000 times, and computing the number of shuffled time segments with amplitude or frequency co-modulation in the high-gamma band, which also contained co-modulation of amplitude or frequency in other frequency bands (theta/alpha, beta, low gamma frequency bands) and assessing whether coincidence rates lied within or outside 95th percentile of the shuffled series.

## 3 Results

**Fig. 2:**
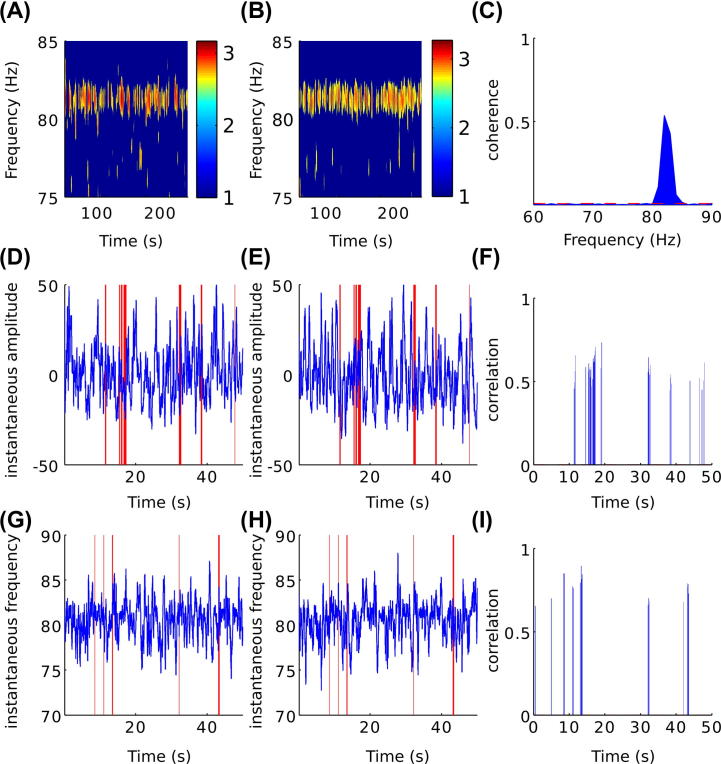
Co-modulation of amplitude envelope and instantaneous frequency in subject 8. (A and B) Log normalized STN LFP power spectrum in the high-gamma band from the left and right STN, respectively. (C) Significant coherence between the left and right STN in the high-gamma band; dotted red line depicts the threshold for coherence set to the 95th percentile of the surrogate dataset. (D and E) Example changes in the amplitude envelope of STN high-gamma oscillations in the left and right STN, respectively. (F) Corresponding significant correlation between amplitude envelope changes. (G and H) Example changes in the instantaneous frequency of STN high-gamma oscillations in the left and right STN, respectively. (I) Corresponding significant correlation between instantaneous frequency changes. Correlated segments are highlighted using red vertical lines in D, E, G and H. In F and I, significance is determined according to the 95th percentile of the corresponding correlation levels derived from the surrogate dataset. (For interpretation of color in this Figure, the reader is referred to the web version of this article.)

All 10 PD subjects had bilateral discrete peaks in the high-gamma frequency band in power spectra of STN-LFPs simultaneously recorded in the ON L-DOPA state. Bilateral STN FTG oscillations were significantly coherent in only three out of the ten cases (*p* ⩽ 0.05; unpaired one-sided *t*-test; subjects 7, 8 and 10; Fig. 2A–C) despite the peak FTG frequency being the same in 5 subjects (Table 2). Four of the subjects demonstrated coherence in the theta/alpha frequency band (*p* ⩽ 0.05; unpaired one-sided *t*-test; subjects 5, 6, 9 and 10) and only one of the subjects demonstrated coherence in the beta band in the ON state (*p* ⩽ 0.05; unpaired one-sided *t*-test; subject 10). None of the subjects demonstrated coherence in the low-gamma band in the ON state (*p* > 0.05; unpaired one-sided *t*-test).

**Table 2:** Peak theta/alpha, beta and FTG frequencies in the left and right STN: In 5/10 subjects, peak FTG frequency was the same across the hemispheres. FTG activity was coherent across hemispheres in cases 7, 8 and 10.

	Peak alpha/theta frequency		Peak beta frequency		Peak FTG frequency	
	Left STN	Right STN	Left STN	Right STN	Left STN	Right STN
1	5 Hz	/	24 Hz	/	74 Hz	74 Hz
2	/	/	26 Hz	25 Hz	72 Hz	71 Hz
3	/	11 Hz	/	17 Hz	69 Hz	65 Hz
4	/	/	/	/	74 Hz	71 Hz
5	9 Hz	/	27 Hz	/	89 Hz	84 Hz
6	/	/	25 Hz	24 Hz	79 Hz	79 Hz
7	/	/	16 Hz	16 Hz	81 Hz	81 Hz
8	/	7 Hz	27 Hz	25 Hz	81 Hz	81 Hz
9	8 Hz	8 Hz	16 Hz	16 Hz	78 Hz	80 Hz
10	11 Hz	10 Hz	25 Hz	/	85 Hz	85 Hz

### 3.1 Bilateral FTG amplitude and frequency co-modulation

**Fig. 3:**
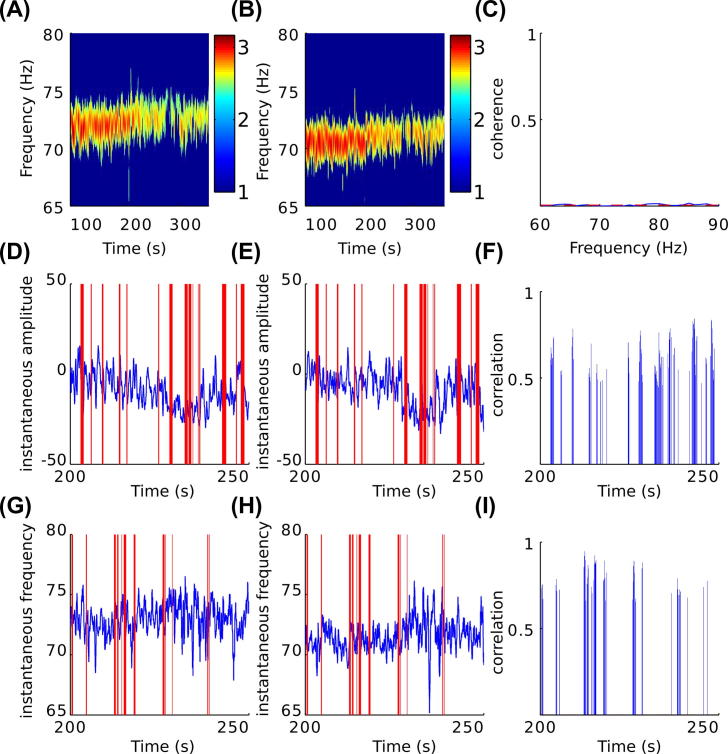
Co-modulation of amplitude envelope and instantaneous frequency in subject 2. (A and B) Log normalized STN LFP power spectrum in the high-gamma band from the left and right STN, respectively. (C) There is no coherence between the left and right STN in the high-gamma band; dotted red line depicts the threshold for coherence set to the 95th percentile of the surrogate dataset. (D and E) Example changes in the amplitude envelope of STN high-gamma oscillations in the left and right STN, respectively. (F) Corresponding significant correlation between amplitude envelope changes. (G and H) Example changes in the instantaneous frequency of STN high-gamma oscillations in the left and right STN, respectively. (I) Corresponding significant correlation between instantaneous frequency changes. Correlated segments are highlighted using red vertical lines in D, E, G and H. In F and I, significance is determined according to the 95th percentile of the corresponding correlation levels derived from the surrogate dataset. (For interpretation of color in this Figure, the reader is referred to the web version of this article.)

**Fig. 4:**
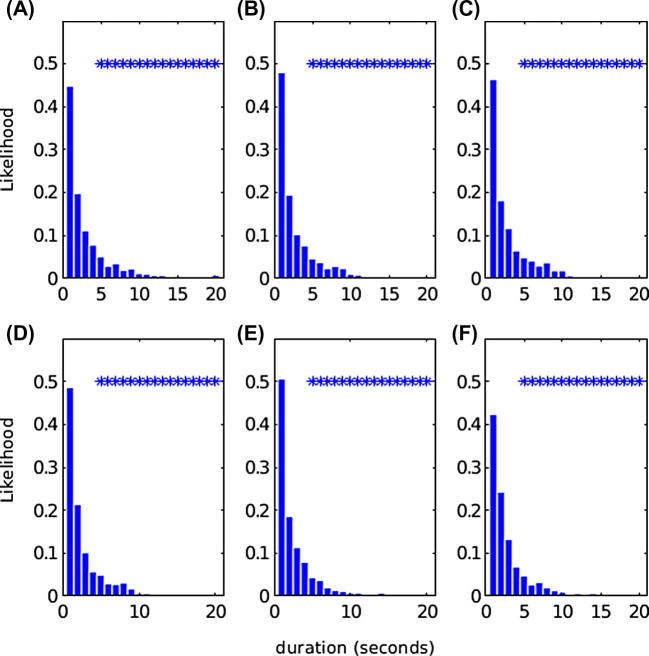
Group data for likelihood of a given duration of co-modulation of the envelopes of amplitude (A–C) and instantaneous frequency (D–F) of bilateral theta/alpha (A: amplitude co-modulation D: frequency co-modulation), beta (B: amplitude co-modulation E: frequency co-modulation) and high-gamma oscillations (C: amplitude co-modulation F: frequency co-modulation). Durations ⩾2 s imply that ⩾2 one-second periods are contiguous and significant. Bins marked with * indicate contiguous durations which are significantly different from those that could occur at random (*p* ⩽ 0.05; unpaired one-sided *t*-test).

We investigated whether the amplitude envelopes or instantaneous frequencies of the STN FTG oscillations were correlated bilaterally (Fig. 2, Fig. 3). On average, 7.4% of each recording showed significant correlation of high-gamma band amplitude-envelopes across the hemispheres (*p* ⩽ 0.05; unpaired one-sided *t*-test) with an average lag of 0 s. These short duration bilateral co-modulations of the amplitude-envelopes could last up to 18 s (Fig. 4). Additionally, significant correlation of the instantaneous frequency of the STN FTG oscillations across the hemispheres was observed with an average lag of 0 s for an average of 8.1% of each recording (*p* ⩽ 0.05; unpaired one-sided *t*-test). Co-modulation of the instantaneous frequencies could last up to 15 s. Bilateral co-modulation of amplitude envelopes and instantaneous frequency did not coincide in time above chance level for any subject.

### 3.2 Bilateral amplitude and frequency co-modulation in other frequency bands

For comparison, we also investigated whether the amplitude envelope or instantaneous frequency of STN theta/alpha (4–14 Hz), beta (15–30 Hz) and low gamma (31–48 Hz) oscillations were correlated bilaterally. The amplitude envelopes of theta/alpha oscillations were significantly correlated across the hemispheres on average 9.7 ± 1.4% of the recording time (*p* ⩽ 0.05; unpaired one-sided *t*-test) with an average lag of 0 s (Table 3), whereas the instantaneous frequency of the STN theta/alpha oscillations was correlated across the two hemispheres on average 7 ± 0.7% of the recording time (*p* ⩽ 0.05; unpaired one-sided *t*-test) with an average lag of 0 s (Table 3). Co-modulation of amplitude and frequency in the theta/alpha frequency band coincided in time.

**Table 3:** Average lag (seconds) and incidence rate of significant bilateral co-modulation of amplitude envelopes and instantaneous frequency in the theta/alpha, beta, low and high gamma frequency bands.

	Theta amplitude		Theta frequency		Beta amplitude		Beta frequency	
	Incidence (%)	Average lag (s)	Incidence (%)	Average lag (s)	Incidence (%)	Average lag (s)	Incidence (%)	Average lag (s)
1	7.4	−0.002	6.8	0	4.35	0.002	3.7	0.006
2	17.5	0.002	10.4	0.002	7.2	0.004	6.2	0
3	4.9	0.01	3.2	−0.012	6.9	0	2	0.006
4	17.7	0	8.2	0	12.5	0	5.3	0.002
5	8.9	0	7	0.002	6.9	0	5	0
6	7.5	0	3.5	0	6.7	0	5.2	0
7	8.6	−0.002	9.4	0.004	5	0.002	3.1	0.002
8	6.2	0	7.8	0	5.7	0	6.9	0
9	6.4	0	5.7	−0.002	16.7	0.004	12	0.004
10	11.4	−0.002	7.6	0	5.9	−0.002	4	0
Average	9.7 ± 1.4	0 ± 0.002	7 ± 0.7	0 ± 0.002	7.8 ± 1.2	0 ± 0.002	5.3 ± 0.9	0.002 ± 0.002

	Low Gamma amplitude		High gamma amplitude		High gamma frequency	
	Incidence (%)	Average lag (s)	Incidence (%)	Average lag (s)	Incidence (%)	Average lag (s)
1	6.1	0	7.6	0	10.8	0
2	7.5	0	14	−0.002	14.7	0
3	4.6	0.002	3.9	0	14.7	0
4	10.3	0	8.9	-0.002	4.9	0
5	5.6	0	6.6	0	5.6	0
6	4.2	0	9.1	0	12.2	0
7	3.1	−0.004	7.7	−0.002	4.7	−0.002
8	5.9	0.002	6.9	0.002	5.4	0
9	5.4	−0.004	5	0.004	4	−0.004
10	3.9	0.002	4.4	−0.002	4.3	−0.004
Average	5.7 ± 0.7	0 ± 0.002	7.4 ± 0.9	0 ± 0.002	8.1 ± 1.4	0 ± 0.002

The amplitude envelopes of beta oscillations were also significantly correlated across the hemispheres on average 7.8 ± 1.2% of each recording (*p* ⩽ 0.05; unpaired one-sided *t*-test; average lag = 0 s) (Table 3); and the beta instantaneous frequency was correlated on average 5.3 ± 0.9% of each recording across the two hemispheres (*p* ⩽ 0.05; unpaired one-sided *t*-test; average lag = 0.002 s) (Table 3).

Amplitude envelopes of low gamma oscillations were significantly correlated across hemispheres on average 5.7 ± 0.7% of each recording (*p* ⩽ 0.05; unpaired one-sided *t*-test) with an average lag of 0 s (Table 3). There were no discrete peaks in power in the low-gamma frequency range therefore it was not possible to investigate instantaneous frequency co-modulation in this frequency band.

### 3.3 Relationship between high-gamma band and other frequency bands

In addition, we studied whether bilateral co-modulation of the amplitude of STN FTG oscillations coincided with bilateral co-modulation of the amplitude of STN beta oscillations. In 10/10 cases, co-modulation of the amplitude of STN FTG oscillations did not occur at the same time with co-modulation of the amplitude of STN beta oscillations (*p* ⩽ 0.05; unpaired one-sided *t*-test). Similarly, co-modulation of the frequency of STN FTG oscillations did not occur at the same time with co-modulation of the frequency of STN beta oscillations (*p* ⩽ 0.05; unpaired one-sided *t*-test). Furthermore, co-modulation of the amplitude or instantaneous frequency of STN FTG oscillations did not coincide with co-modulation in the theta/alpha band (4–14 Hz) or those in the low-gamma frequency band (*p* ⩽ 0.05; unpaired one-sided *t*-test).

## 4 Discussion

We have shown that there is co-modulation of FTG oscillations in the STN of the two hemispheres in patients with PD. This could take the form of amplitude co-modulation, frequency co-modulation, combined but dissociated in time amplitude and frequency co-modulation, and, in three patients, cotemporous amplitude and phase co-modulation implied by coherence. Co-modulation was truly synchronised, with mean temporal differences of zero milliseconds between sides. Anatomical evidence for direct connectivity between the two STNs is lacking in the primate (Carpenter and Strominger, 1967, Carpenter et al., 1981), although limited activity in the contralateral STN can be evoked at very short-latency by stimulation of the ipsilateral nucleus, possibly through stimulation of fibres of passage (Brun et al., 2012, Walker et al., 2011). The bulk of modulatory effects of stimulation of one STN upon the other are of much longer latency than the temporal difference between STNs reported here, and are compatible with indirect communication between the two STNs (Brun et al., 2012). Thus our findings suggest one or more bilateral common inputs to the two STN that modulate FTG activities across the hemispheres more-or-less simultaneously. The simplest explanation for such common input would be that linked to voluntary movement, which in PD can be associated with bilateral (albeit greater contra-laterally) amplitude increases in FTG, although bilateral frequency modulation has not been previously reported (Androulidakis et al., 2007a, Cassidy et al., 2002). However, our recordings were made with patients at rest. Furthermore, although voluntary movements are associated with a phasic reciprocal change in the high-gamma and beta bands, we found FTG co-modulation episodes to be isolated, and more prolonged than those observed with voluntary movement (Androulidakis et al., 2007a, Cassidy et al., 2002).

Could co-modulation of FTG be related to concurrent dyskinesias, given that six of the subjects had symptomatic dyskinesias prior to the operation? Subtle dyskinesias may have been missed during the recordings, but the relationship of this involuntary movement to FTG is unclear. Fogelson et al. (2005) related dyskinesias to FTG activity, but only in the presence of reciprocal changes in beta activity. The latter were missing in the current study. Other authors have suggested that dyskinesias relate to theta/alpha power rather than FTG activity (Alegre et al., 2012, Alonso-Frech et al., 2006, Rodriguez-Oroz et al., 2011). Bilateral changes in amplitude envelope and instantaneous frequency in the alpha/theta frequency band were not present during co-modulation of amplitude or instantaneous frequency in the high-gamma frequency band.

Equally, could co-modulation of FTG be related to concurrent tremor, given that three of the subjects had symptomatic tremor prior to the operation? Subtle rest tremor may have been missed by visual inspection during the recordings. However, there are no reports relating the presence or character of FTG (over 60–90 Hz) to residual rest tremor in the ON medication state, although one study has detailed a relationship between rest tremor and LFP power in the contra-lateral STN over a lower gamma frequency range from 35 to 55 Hz (Weinberger et al., 2009). FTG co-modulation did not coincide with low-gamma co-modulation. In the present study although two of the three cases with bilateral FTG coherence had symptomatic tremor, including one case with tremor-dominant PD, the other patient with tremor-dominant PD, did not have bilateral FTG coherence.

Another event associated with bilateral amplitude co-modulation in FTG is arousal, as elicited by startling auditory stimuli or as occurring spontaneously in REM sleep (Kempf et al., 2009). Related to this, FTG is known to be suppressed during drowsiness (Brown et al., 2001). Thus the relatively prolonged co-modulation across the hemispheres shown here could be due to spontaneous fluctuations in arousal (Jenkinson et al., 2013). If so, then these fluctuations were subtle enough not to manifest clinically, although subliminal variations in arousal (or the related phenomenon of vigilance) have long been believed to be responsible for fluctuations in performance measures like reaction time (Lansing et al., 1959). Indeed, a correlation has recently been reported between high-gamma levels in the thalamus prior to imperative cues and subsequent reaction time (Brücke et al., 2013). A link with arousal does not preclude further more marked changes in FTG that are more specifically related to movement and which tend to be more lateralised (Androulidakis et al., 2007a, Brücke et al., 2008).

If co-modulation of FTG at rest relates to minor fluctuations in arousal then which pathways may mediate these effects? Projections from the cerebral cortex are unlikely drivers, as FTG in the STN drives (phase-leads) rather than is driven by cortical activity (Lalo et al., 2008, Litvak et al., 2012, Williams et al., 2002). Perhaps more likely is the possibility that high-gamma is bilaterally modulated by the ascending reticular activating system, including the pedunculopontine nucleus (Jenkinson et al., 2009, Pahapill and Lozano, 2000), either directly or via the non-specific nuclei of the thalamus (Sadikot et al., 1992).

In order to maximise our ability to track variation in the amplitude and frequency of the FTG we opted to assess PD patients after treatment with dopaminergic medication including L-DOPA, as FTG is more prominent in this state (Alegre et al., 2005, Alonso-Frech et al., 2006, Androulidakis et al., 2007a, Brown et al., 2001, Cassidy et al., 2002, Devos et al., 2006, Fogelson et al., 2005). This, however, may account for the relative lack of co-modulation and coherence of beta activity across the hemispheres in our patient cohort compared to recordings made off L-DOPA (de Solages et al., 2010). Dopaminergic treatment suppresses beta activity and may also promote its reactivity and lateralisation (Androulidakis et al., 2007b). In contrast, beta activity tends to be bilaterally coherent (show amplitude and phase covariation) in patients withdrawn from antiparkinsonian medication (de Solages et al., 2010). In four out of 10 subjects we also observed significant coherence in the theta/alpha frequency band despite lack of discrete theta/alpha oscillations as evidenced by a peak in this frequency band.

Another factor that might have contributed to the relative sparseness of cross-hemisphere co-modulation and coherence is the possibility of a stun effect in our post-operative recordings. This can attenuate LFP activity in the beta frequency band (Chen et al., 2006), but little, if anything, is known about its effects on FTG or on cross-hemisphere coupling.

Although we recorded FTG in the STN of patients with PD, this activity is by no means disease specific, having also been reported in patients with essential tremor, dystonia and myoclonic epilepsy (Kempf et al., 2009). Moreover, FTG is likely seen throughout the BG, having been found in recordings from the thalamus, globus pallidus interna (GPi) and STN (Alegre et al., 2012, Alonso-Frech et al., 2006, Androulidakis et al., 2007b, Brown et al., 2001, Cassidy et al., 2002, Devos et al., 2006, Fogelson et al., 2006, Kempf et al., 2009, López-Azcárate et al., 2010, Pogosyan et al., 2006, Trottenberg et al., 2006, Williams et al., 2002). These observations lead to speculation that the FTG may be an essentially physiological, possibly arousal related, phenomenon.

Finally, the temporal independence of co-modulations in high-gamma frequency and amplitude, and of these and amplitude and frequency co-modulation in the theta/alpha, beta and low-gamma bands supports the idea that synchronisation at different frequencies could provide a means of functionally segregating processing streams that may then be multiplexed in the motor system (Fogelson et al., 2006). Furthermore, it allows for the relatively independent organisation of processing through amplitude and frequency modulation of on-going oscillations (Foffani et al., 2005). Whether these multiple dimensions of information carrying capacity are actually utilised deserves further investigation.
